# Sirtuin Lipoamidase Activity Is Conserved in Bacteria as a Regulator of Metabolic Enzyme Complexes

**DOI:** 10.1128/mBio.01096-17

**Published:** 2017-09-12

**Authors:** Elizabeth A. Rowland, Todd M. Greco, Caroline K. Snowden, Anne L. McCabe, Thomas J. Silhavy, Ileana M. Cristea

**Affiliations:** Department of Molecular Biology, Lewis Thomas Laboratory, Princeton University, Princeton, New Jersey, USA; Harvard Medical School

**Keywords:** CobB, SIRT, SRM, acylation, delipoylation, mass spectrometry, proteomics, selected reaction monitoring, sirtuin

## Abstract

Lipoic acid is an essential metabolic cofactor added as a posttranslational modification on several multimeric enzyme complexes. These protein complexes, evolutionarily conserved from bacteria to humans, are core regulators of cellular metabolism. While the multistep enzymatic process of adding lipoyl modifications has been well characterized in *Escherichia coli*, the enzyme required for the removal of these lipoyl moieties (i.e., a lipoamidase or delipoylase) has not yet been identified. Here, we describe our discovery of sirtuins as lipoamidases in bacteria and establish their conserved substrates. Specifically, by using a series of knockout, overexpression, biochemical, *in vitro*, proteomic, and functional assays, we determined the substrates of sirtuin CobB in *E. coli* as components of the pyruvate dehydrogenase (PDH), α-ketoglutarate dehydrogenase (KDH), and glycine cleavage (GCV) complexes. *In vitro* assays provided direct evidence for this specific CobB activity and its NAD^+^ dependence, a signature of all sirtuins. By designing a targeted quantitative mass spectrometry method, we further measured sirtuin-dependent, site-specific lipoylation on these substrates. The biological significance of CobB-modulated lipoylation was next established by its inhibition of both PDH and KDH activities. By restricting the carbon sources available to *E. coli*, we demonstrated that CobB regulates PDH and KDH under several growth conditions. Additionally, we found that SrtN, the sirtuin homolog in Gram-positive *Bacillus subtilis*, can also act as a lipoamidase. By demonstrating the evolutionary conservation of lipoamidase activity across sirtuin homologs, along with the conservation of common substrates, this work emphasizes the significance of protein lipoylation in regulating central metabolic processes.

## IMPORTANCE

Here, we demonstrate that sirtuin lipoamidase activity exists in both Gram-positive and Gram-negative bacteria and establishing its conservation from bacteria to humans. Specifically, we discovered that CobB and SrtN act as lipoamidases in *E. coli* and *B. subtilis*, respectively. Intriguingly, not only is this sirtuin enzymatic activity conserved, but also the lipoylated substrates and functions are conserved, as bacterial sirtuins negatively regulate the lipoylation levels and activities of PDH and KDH. Considering that PDH and KDH regulate two carbon entry points into the tricarboxylic acid cycle, our finding highlights lipoylation as a conserved molecular toggle that regulates central metabolic pathways. Indeed, our findings from tests in which we limited nutrient availability support this. Furthermore, this study illustrates how the integration of technologies from different disciplines provides avenues to uncover enzymatic activities at the core of cellular metabolism regulation.

## INTRODUCTION

**FIG 1:**
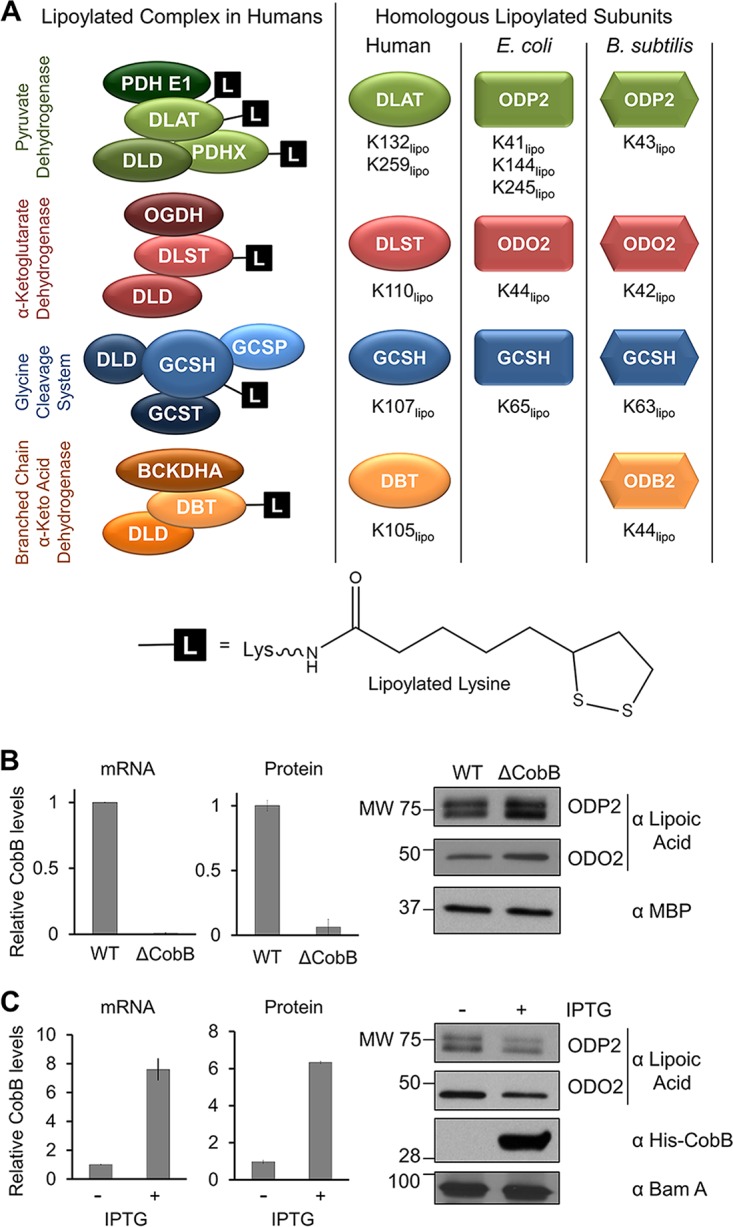
CobB levels are inversely correlated with protein lipoylation levels in *E. coli*. (A) Lipoylated protein complexes are conserved across humans, *E. coli*, and *B. subtilis*; L depicts lipoylation. Homologous complex subunits and modified lysine residues (K_lipo_) are indicated. (B) Assessment of CobB mRNA levels via qPCR (left), of CobB protein levels via MS (middle), and of lipoyl levels via Western blotting (right) in the Δ*cobB* strain relative to WT. MBP was used as a loading control. SRM-MS/MS quantification (middle) showed relative levels of a representative unmodified CobB peptide, KYYGPASQVVPE. The detected signal in the Δ*cobB* strain was at the level of noise in the MS analysis (i.e., undetected CobB). (C) Assessment of CobB mRNA levels, CobB protein levels, and lipoyl levels in the +IPTG group relative to the uninduced strain, as shown in panel B. The SRM-MS/MS detected signal in the uninduced cells (middle) was derived from combined MS noise and leaky plasmid expression. IPTG induction of His-CobB was seen using anti-His antibody; BamA was used as a loading control. Error bars represent standard deviations.

Lipoic acid is an evolutionarily conserved essential metabolic cofactor (1–3). Known as lipoamide in its oxidized form and dihydrolipoamide in its reduced form, the lipoyl cofactor is required for the covalent modification of lysine residues on proteins, a modification known as lipoylation. Specifically, this modification has been identified on proteins critical for the regulation of cellular metabolism. In humans, four metabolic multimeric enzyme complexes are known to be lipoylated: pyruvate dehydrogenase (PDH), α-ketoglutarate dehydrogenase (KDH), branched-chain α-keto acid dehydrogenase (BCDH), and the glycine cleavage complex (GCV) (2, 3) (Fig. 1A). The lipoylated residues are present in two subunits of the human PDH complex, the dihydrolipoyllysine residue acetyltransferase (DLAT) and the pyruvate dehydrogenase protein X component (PDHX). Human KDH, BCDH, and GCV contain one lipoylated subunit each: the dihydrolipoyllysine residue succinyltransferase component of KDH (DLST), the lipoamide acyltransferase component of BCDH (DBT), and the glycine cleavage complex H protein (GCSH), respectively. In each complex, the lipoyl modification is thought of as a “swinging arm”; the reduction of lipoamide allows for the capture and subsequent presentation of the substrate intermediate during the complex’s enzymatic process (2, 3). This modification can also contribute to the structural integrity of these multimeric complexes (3). Strikingly, these specific lipoylated proteins are conserved from bacteria to mammals, pointing to a likely critical role for this modification in regulating the function of these complexes, although this remains to be proven. Gram-negative and Gram-positive species of bacteria have homologous complexes that are modified by lipoylation (Fig. 1A). In *Escherichia coli*, three of the lipoylated complexes in humans are conserved: PDH, KDH, and GCV. *Bacillus subtilis* also has a conserved lipoylated BCDH, in addition to the three complexes found in both humans and *E. coli* (2).

Lipoamidase activity, which removes lipoyl modifications (i.e., delipoylation), was detected in the 1950s (1, 4, 5), and to this day, the only enzyme found to have this function in bacteria is a protein named lipoamidase (Lpa) in *Enterococcus faecalis* (6, 7). The knowledge of delipoylating enzymes was recently expanded when we discovered mitochondrial sirtuin 4 (SIRT4) as a mammalian lipoamidase (8). We further demonstrated that SIRT4-mediated lipoyl levels regulated PDH enzymatic activity (8). This finding adds to the knowledge that mitochondrial sirtuins (SIRT3, -4, and -5) act as critical sensors and regulators of metabolic pathways by rapidly responding to changes in their environment. Furthermore, the identification of lipoamidase activity adds to the growing range of sirtuin enzymatic activities. While originally thought to only act as NAD^+^-dependent deacetylases, recent studies have identified several additional enzymatic activities for mitochondrial sirtuins, including ADP-ribosylation (for SIRT4) and desuccinylation and demalonylation (for SIRT5) (8–12). Altogether, these activities allow mitochondrial SIRTs to regulate diverse substrates with functions in critical metabolic pathways, including the tricarboxylic acid (TCA) cycle, fatty acid oxidation, the urea cycle, and glycolysis (8–15).

The ability of SIRTs to perform multiple enzymatic functions has also been demonstrated in bacteria. Both CobB of *E. coli* and *Salmonella enterica* and SrtN of *B. subtilis* have been characterized as NAD^+^-dependent deacetylases that activate the conversion of acetate to acetyl coenzyme A (CoA) by acetyl-CoA synthetase (16–18). Additionally, CobB has been shown to act as a desuccinylase, similar to human mitochondrial SIRT5 (19). Based on sequence homology, CobB and SrtN most closely resemble mitochondrial SIRT4 and SIRT5 (17, 18, 20). Recently, CobB was also implicated in catalyzing dehomocysteinylation and was proposed to have a potential role in removal of propionylation (21, 22). These results point toward a broader role for CobB than just as a deacetylase. Similar to some human sirtuins, CobB may act as a more versatile deacylase.

Given our previous findings on human SIRT4 and the fact that a lipoamidase enzyme has not yet been discovered in *E. coli* and *B. subtilis*, we investigated whether this sirtuin enzymatic function is conserved in bacteria. By using a series of knockout, overexpression, *in vitro*, and biochemical assays, we demonstrated the conservation of both lipoamidase activity and common substrates for CobB in *E. coli*. We further developed a targeted quantitative mass spectrometry (MS) approach to monitor sirtuin-dependent lipoylation on specific lysines in PDH and GCV. The biological significance of bacterial sirtuin-modulated lipoylation was established by the ability of CobB to regulate PDH and KDH activities when challenged under various growth conditions. Altogether, our results have led to the discovery of two previously unreported bacterial lipoamidases, expanding the current understanding of the critical and conserved roles of lipoylation in regulating cellular metabolism.

## RESULTS

### Sirtuin CobB levels are inversely correlated with lipoylation levels of dehydrogenase complexes in *E. coli.*

To determine whether CobB acts as a lipoamidase, we first investigated the impact of CobB protein levels on whole-cell lipoylation. For this, we compared wild-type *E. coli* (MC4100-wt) with a Δ*cobB* deletion strain (*cobB* knockout in MC4100 cells) that we previously generated (23). CobB depletion was confirmed at the mRNA level by using quantitative PCR (qPCR) (Fig. 1B, left). As an antibody against CobB was not available, we designed a targeted MS approach based on selective reaction monitoring (SRM), which allowed us to monitor the levels of CobB peptides and confirm decreased protein levels in Δ*cobB* strain cells (Fig. 1B, center). We next monitored global levels of protein lipoylation in these cells by using Western blotting (WB). CobB knockout resulted in increased lipoylation of multiple proteins within the cell which, based on their masses, we predicted to be the PDH subunit ODP2 (homolog of human DLAT) and the KDH subunit ODO2 (homolog of human DLST) (Fig. 1B, right). To further confirm these results, we next performed the reciprocal experiment by monitoring the effect of CobB overexpression. Using an isopropyl-β-d-thiogalactopyranoside (IPTG)-inducible plasmid (23, 24), we overexpressed His-CobB in the Δ*cobB* strain (Fig. 1C), thereby limiting potential difficulties with differentiating the effects from mixed populations of CobB (endogenous and exogenous His-CobB). CobB overexpression was confirmed by both qPCR and quantitative MS, as described above. Successful induction of the plasmid was also seen by WB when we probed for the His tag on His-CobB. Increased CobB levels resulted in reduced lipoylation for the predicted ODP2 and ODO2 (Fig. 1C, right). Altogether, our results demonstrated that total lipoyl levels in cell lysates are inversely correlated with CobB protein levels, supporting our further investigation of a possible CobB lipoamidase activity.

### *In vitro* assays provide direct evidence of CobB NAD^+^-dependent lipoamidase activity.

**FIG 2:**
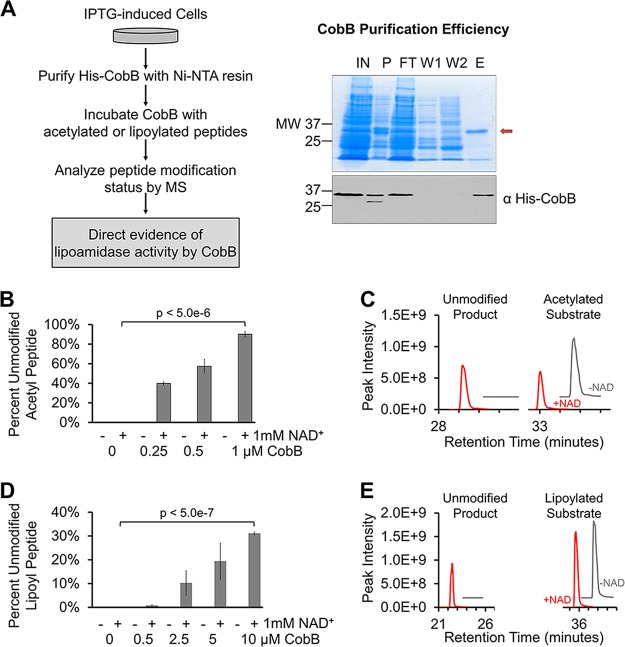
*In vitro* evidence for CobB lipoamidase activity. (A) Workflow developed to purify His-CobB and analyze enzymatic activity *in vitro* (left). Isolation of His-CobB, as shown on a protein gel (right, top) and Western blotting (right, bottom). His-CobB is indicated (red arrow) on the protein gel. His-CobB was seen on the Western blot when we used anti-His antibody. Lane labels: IN, input; P, pellet (insoluble fraction); FT, flowthrough; 1, wash 1; 2, wash 2; E, elution. (B) Percentage of unmodified peptide present after deacetylation of synthetic acetyl-peptide after 1-h incubations with various concentrations of CobB, with and without 1 mM NAD^+^ (*n* = 3). Synthetic posttranslationally modified H3K9 peptides were used in all *in vitro* assays. (C) Representative extracted ion chromatograms showing levels of resulting unmodified product (left) and remaining acetylated substrate (right) after 1-h incubation with 0.5 µM CobB. Chromatograms from reactions without NAD (-NAD) are offset for clarity (*x* axis, +2; *y* axis, +2e8). (D) Percentage of unmodified peptide present after removal of lipoylation from synthetic lipoyl-peptide after 1-h incubation with various concentrations of CobB, with and without 1 mM NAD^+^ (*n* = 3). (E) Representative extracted ion chromatograms showing unmodified product (left) and remaining lipoylated substrate (right) after 1 h with 10 µM CobB. Chromatograms from reactions without NAD are offset for clarity (*x* axis, +3; *y* axis, +2e8). Error bars represent standard deviations.

To provide direct evidence of lipoyl removal by CobB, we next performed an *in vitro* assay using purified CobB (Fig. 2A, left). His-CobB was purified via the His tag using nickel-nitrilotriacetic acid (Ni-NTA)–agarose beads (Fig. 2A, right). The eluted fraction (Fig. 2A, lane E) was then dialyzed against exchange buffers to remove the low-molecular-weight proteins seen in the gel. Various concentrations of purified CobB were incubated for 1 h with synthetic peptides containing either an acetylated or lipoylated lysine residue, in the presence or absence of NAD^+^. Each *in vitro* reaction mixture was analyzed by MS to quantify the levels of the resulting unmodified peptides. As a positive control, to confirm that CobB retained its enzymatic activity during the purification process and to validate our reaction conditions, we first tested the ability of CobB to remove the acetyl modification. We saw robust CobB concentration-dependent and NAD^+^-dependent deacetylase activity (Fig. 2B and C). When incubated with the lipoylated peptide, CobB was also able to remove the lipoyl modification, and this activity was again NAD^+^ dependent (Fig. 2D and E). Although more CobB was required to remove similar levels of lipoylation compared to acetyl groups, this activity still occurred in a CobB concentration-dependent manner. Furthermore, when we extended the reaction time while keeping the concentration of CobB constant, we saw that CobB continued to remove lipoyl groups throughout a 3-h incubation period (see Fig. S1 in the supplemental material). This suggested that CobB lipoamidase activity was not saturated after the 1-h incubation (Fig. 2D). Together, these data provide direct evidence that CobB is able to remove lysine lipoylation *in vitro*.

10.1128/mBio.01096-17.1 FIG S1  CobB hydrolyzes acetyl- and lipoyl-modified synthetic peptides *in vitro*. (A) CobB at 0.25 µM was incubated with 10 µM acetyl-peptides with 1 mM NAD^+^ at 37°C for the times indicated (*n* = 1). (B) CobB at 5 μM was incubated with 10 µM lipoyl-peptides with 1 mM NAD^+^ at 37°C for the times indicated (*n* = 1). Synthetic posttranslationally modified H3K9 peptides were used in these assays. Download FIG S1, TIF file, 1 MB. Copyright © 2017 Rowland et al. 2017 Rowland et al. This content is distributed under the terms of the Creative Commons Attribution 4.0 International license.

### Dehydrogenase and glycine cleavage complexes are substrates of CobB lipoamidase activity.

**FIG 3:**
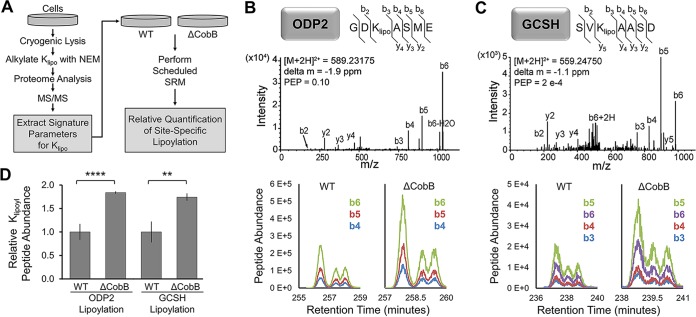
Dehydrogenase and glycine cleavage complexes are substrates of sirtuin CobB lipoamidase activity in *E. coli*. (A) Targeted mass spectrometry workflow developed for K_lipo_ quantification by SRM-MS. (B and C) Representative MS/MS spectra of lipoylated peptides in ODP2 and GCSH; the detected b and y ions are indicated. Chromatograms of peptides in WT and the Δ*cobB* deletion strain are shown below. Each curve shows a unique b ion for ODP2 and GCSH peptides. (D) SRM relative quantification of lipoylation in ODP2 and GCSH in WT and Δ*cobB* mutant *E. coli* (*n* = 3), **, *P* < 0.01; ****, *P* < 0.001. Error bars represent standard deviations.

Having confirmed CobB *in vitro* lipoamidase activity, we next investigated its biological substrates. While the WB results (Fig. 1) showed CobB-dependent alterations in protein lipoylation levels, these analyses did not confirm the substrate identities, nor did they provide accurate quantification or site-specific information about the lipoylated residues. Furthermore, GCV is expected to be lipoylated in *E. coli*, but this was not visible by WB, likely due to its low molecular mass (13 kDa). To address these issues, we next designed a targeted SRM-tandem MS (SRM-MS/MS) approach to quantify protein lipoylation at specific lysine residues (Fig. 3A). Before performing this quantification, we confirmed that the predicted lipoylated peptides could be detected in whole-cell lysates via the following qualitative approach (Fig. 3A, workflow on left side): (i) reduction of disulfide bonds, including the lipoamide dithiolane ring, (ii) alkylation of lipoylated lysines (K_lipo_), (iii) data-dependent MS analysis of the *E. coli* proteome following digestion with GluC, and (iv) MS/MS confirmation of lipoyl-peptide identification for putative CobB substrates. Next, the unique lipoyl-peptide parameters (mass/charge, retention time, and prominent fragment ions) derived from these analyses were used to generate a targeted quantification (SRM-based) method for the relative quantification of lipoylation levels (Fig. 3A, workflow on right side).

Given our prior findings for human SIRT4 (8) and the *E. coli* WB results (Fig. 1), we focused this analysis on PDH and GCV. PDH analysis could point to a conserved substrate for this sirtuin, and GCV, which was not visible by WB, could indicate a possible additional substrate. Indeed, using the MS/MS confirmation approach (Fig. 3A, workflow on the left), we identified the expected K_lipo_ sites on peptides from ODP2 (PDH subunit) and GCSH (GCV subunit) in whole-cell lysates (Fig. 3B and C, top). Importantly, these results provided information about signature peptide fragment ions for ODP2 (b4, B5, and b6) and GCSH (b3, b4, B5, and b6) (Fig. 3B and C, chromatograms at panel bottoms). The impact of CobB deletion on lipoylated peptide abundance was quantified by measuring the integrated intensities of these signature fragment ions (Fig. 3D). In conclusion, this SRM analysis provided quantitative evidence that in Δ*cobB* strain cells there was an increase of K_lipo_ on both PDH and GCV. Together with the WB results, these analyses demonstrate that the lipoylation levels on ODP2 and GCSH are modulated by CobB levels.

### CobB interacts with lipoylated metabolic complexes *in vivo.*

**FIG 4:**
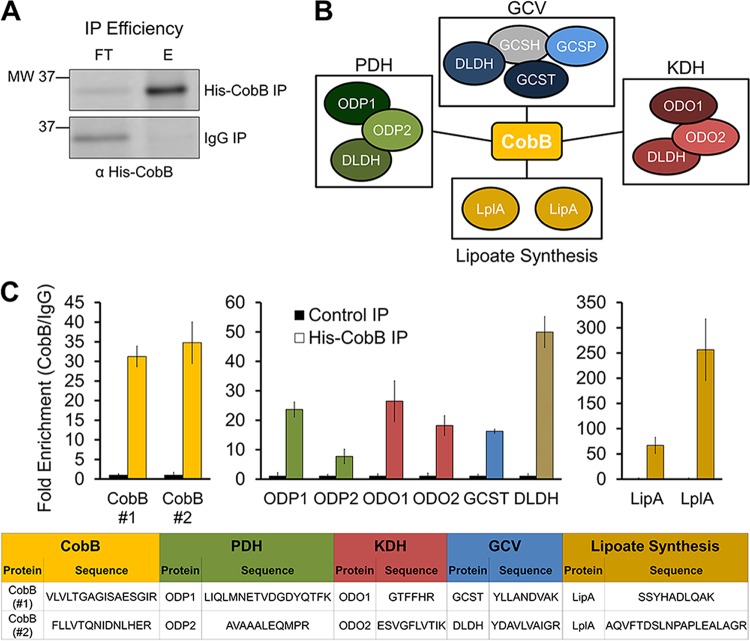
CobB interacts with dehydrogenase and glycine cleavage complexes *in vivo*. (A) IP efficiency for the Western blot, in which His-CobB was detected by using anti-His antibody. Lane labels: FT, flowthroughs; E, elutions from anti-His-CobB and control anti-IgG IPs. (B) Functional protein interaction network of CobB, as determined by data-dependent MS analysis. A gray shape indicates interaction not detected by MS. (C) Fold enrichment of representative peptides from the subunits identified in panel B, as measured by targeted quantitative MS. The quantified peptide sequences are listed in the table portion of the figure. Error bars represent standard deviations.

To further establish that CobB can associate with these substrates *in vivo*, we performed an immunoaffinity purification (IP) experiment, in which His-CobB was isolated via the His tag. CobB was isolated using anti-His antibody and protein A/G magnetic beads. The proteins interacting with CobB were identified following trypsin digestion and data-dependent MS analysis. Using the same cell lysates, anti-IgG antibody conjugated to protein A/G beads acted as the control IP. The efficiency of CobB isolation was confirmed by WB (Fig. 4A). In agreement with our lipoylation analyses (Fig. 1 and 3), we found that CobB associates with ODP2 and ODO2, as well as with all the remaining subunits of the PDH, KDH, and GCV complexes (Fig. 4B; Table S1). GCSH was not identified in our IP, likely due to its considerably smaller mass (13 kDa) and fewer potential tryptic peptides than the other complex components. Additionally, we found CobB interactions with lipoate synthesis proteins, LipA and LplA; these two proteins have roles in lipoate synthesis and scavenging in *E. coli* (2), modifying the same proteins we determined to be CobB substrates. To further confirm these interactions and more accurately quantify their enrichment in the CobB IP relative to the control IP, we designed an additional targeted quantitative MS method to specifically measure peptide abundance from these proteins in IPs from three biological replicates (Fig. 4C; Table S1). The signature peptides quantified are listed in Fig. 4C (bottom). In agreement with our initial data-dependent MS analysis (Fig. 4B; Table S1), this targeted MS quantification confirmed the isolation of CobB (Fig. 4C, left) and its specific interactions with PDH, KDH, and GCV subunits (Fig. 4C, middle), as well as with lipoate synthesis proteins (Fig. 4C, right). Therefore, our interaction study demonstrated *in vivo* associations between CobB and these dehydrogenase and glycine cleavage complexes, further supporting the function of CobB as a lipoamidase in *E. coli*.

10.1128/mBio.01096-17.2 TABLE S1  Data-dependent MS analysis of metabolic complexes in His-CobB IPs. The isolations of His-CobB and IgG were performed, followed by analysis of the subunits of metabolic complexes found in His-CobB and IgG IPs, as represented in Figure 4A. Spectral counts are provided (x, not detected). Download TABLE S1, XLSX file, 0.01 MB. Copyright © 2017 Rowland et al. 2017 Rowland et al. This content is distributed under the terms of the Creative Commons Attribution 4.0 International license.

### CobB and SrtN levels inhibit the activity of dehydrogenase complexes in *E. coli* and *B. subtilis*, respectively.

**FIG 5:**
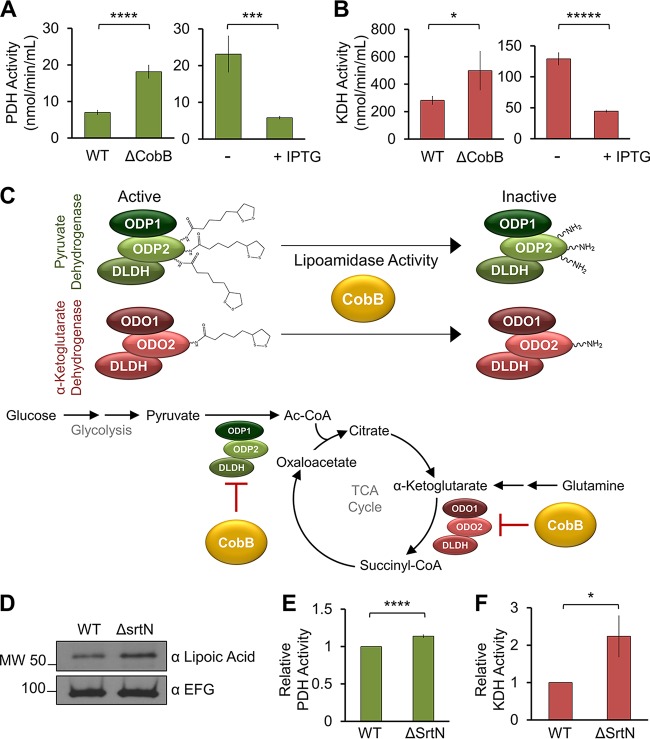
Sirtuins inhibit the activities of metabolic complexes in *E. coli* and *B. subtilis*. (A) Absolute PDH activity measured in WT and the Δ*cobB* deleetion mutant and in IPTG-treated and untreated cells (*n* = 3). ***, *P* < 0.005; ****, *P* < 0.001. (B) Absolute KDH activity measured as for panel A (*n* = 3), except for the Δ*cobB* strain (*n* = 2). *, *P* < 0.05; *****, *P* < 0.0005. (C, top) Proposed CobB-mediated lysine delipoylation reaction and resulting inhibition of complex activity. (Bottom) Proposed metabolic pathways under sirtuin regulation by lipoamidase activity. (D) Assessment of lipoyl levels via Western blotting with a Δ*srtN* deletion mutant relative to WT *B. subtilis*. EFG was used as a loading control. (E) Relative PDH activity measured in WT and Δ*srtN* mutant strain (*n* = 3). ****, *P* < 0.001. (F) Relative KDH activity as measured for panel (E (*n* = 3). *, *P* < 0.05. Error bars represent standard deviations.

Having established PDH as a substrate of CobB, we next sought to determine the biological significance of this lipoamidase activity. A colorimetric assay was used to determine absolute PDH activity in *E. coli* cell lysates from cells grown in LB broth (Miller). CobB deletion resulted in a significant increase in PDH activity compared to WT cells (Fig. 5A, left). In agreement with this observation, CobB overexpression (via IPTG induction) resulted in a significant decline in PDH activity relative to that in the uninduced strain (Fig. 5A, right). Therefore, CobB levels regulate both the lipoylation status and the activity of PDH in *E. coli*, suggesting that sirtuin regulation of PDH is a function conserved in both humans (8) and bacteria.

As our WB results suggested that the next-most-prominent cellular lipoylation is present on the ODO2 subunit, we next assessed the impact of CobB levels on KDH activity. Our results showed a similar trend for the CobB-mediated regulation of KDH as the one we observed for PDH activity. CobB knockout led to increased KDH activity, while increased CobB levels reduced activity (Fig. 5B). This finding revealed a second point of control for CobB to regulate carbon entry into the TCA cycle, indicating that via its lipoamidase activity CobB has the opportunity to modulate diverse cellular metabolic pathways (Fig. 5C). Altogether, our results demonstrate that CobB can directly act on lipoylated lysine residues *in vitro* (Fig. 2) and interact with (Fig. 4), remove lipoylation from (Fig. 3), and inhibit the activity of (Fig. 5) dehydrogenase metabolic complexes *in vivo*.

To further demonstrate the evolutionary conservation of SIRT4 lipoamidase activity in bacteria, we next studied SrtN, the sirtuin homolog in Gram-positive *B. subtilis* (18). By assessing global cellular lipoylation levels via WB, we observed increased lipoylation in a SrtN deletion strain (Δ*srtN* strain BD6189), compared to the WT strain (IS75 strain [25]) (Fig. 5D). We then studied the impact of depleted SrtN levels on PDH and KDH activities. We found that reduced sirtuin levels led to increased KDH activity, as well as a moderate increase in PDH activity (Fig. 5E and F). These results suggest that SrtN may represent a sirtuin lipoamidase in *B. subtilis*.

### The extent of PDH and KDH regulation by CobB is dependent on nutrient availability.

**FIG 6:**
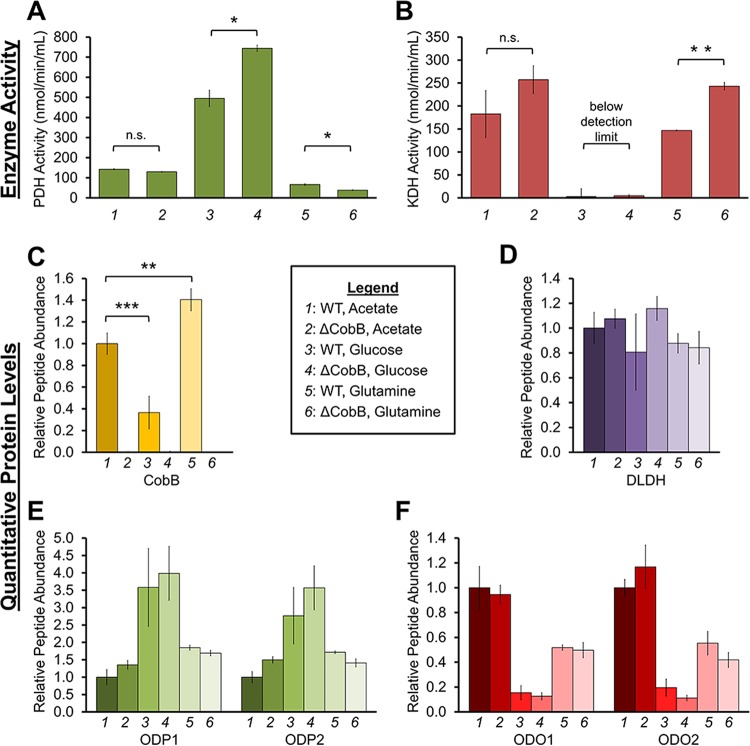
CobB regulates PDH and KDH when cells are challenged under different growth conditions. (A) Absolute PDH activity measured in WT and Δ*cobB* mutant cells when cultured in 0.4% acetate, 1% glucose, or 0.4% glutamine (*n* = 2). n.s., not significant; *, *P* < 0.05. (B) Absolute KDH activity, as measured in panel A (*n* = 2). **, *P* < 0.01. (C to F) Relative peptide quantification by targeted MS for CobB (C) and subunits of PDH (D and E) and KDH (D and F) for cells grown as described for panels A and B (*n* = 3). Error bars represent standard deviations.

Having determined that CobB regulates the activities of PDH and KDH, two complexes positioned at carbon entry points into the TCA cycle, we next investigated whether this regulation is in response to nutrient availability. For this, we evaluated three nutrient sources that enter the TCA cycle via distinct pathways: (i) acetate, which enters downstream of PDH as acetyl-CoA, (ii) glucose, which enters as pyruvate before being converted to acetyl-CoA by PDH, and (iii) glutamine, which enters as α-ketoglutarate that is then converted to succinyl-CoA by KDH. WT and ΔcobB cells were grown in minimal media supplemented with 0.4% acetate, 1% glucose, or 0.4% glutamine as the sole carbon source (nutrient levels were those described in reference 26), and resulting PDH and KDH activities were measured (Fig. 6A and B). To determine whether the measured complex activities are driven by changes in their protein levels, we also performed a targeted MS analysis to quantify CobB, PDH, and KDH protein abundances under the same acetate, glucose, or glutamine growth conditions (Fig. 6C to F). DLDH is a common subunit to PDH, KDH, and GCV, and therefore its levels illustrate the summation of the abundances of all three protein complexes. Peptides from ODP1 and ODP2 provide specific information about the abundance of PDH, while ODO1 and ODO2 peptides inform us of KDH protein levels.

PDH activity was elevated in glucose-fed cells relative to cells grown in either acetate or glutamine (Fig. 6A). This finding correlated with both previous reports (27) and our own observation that glucose also induced PDH protein levels (Fig. 6E). Furthermore, glucose also led to a significant decrease in CobB protein levels (Fig. 6C), which is, to our knowledge, a phenomenon not previously reported. Therefore, the elevated PDH activity seems driven by a combination of both decreased CobB levels and increased PDH protein levels. Additionally, CobB maintained its regulatory ability over PDH in glucose-treated cells, as CobB deletion led to an even further increase in PDH activity (Fig. 6A, compare lanes 3 and 4). This significant additional increase in PDH activity was not due to matching changes in PDH protein levels (Fig. 6E, compare lanes 3 and 4). In contrast to the glucose condition, CobB deletion did not impact PDH activity in cells grown with acetate or glutamine. This was expected, as acetate and glutamine are not incorporated into the TCA cycle via PDH. Additionally, given this bypass of PDH, acetate conditions have been shown to downregulate PDH gene expression (28). Altogether, our results support the prior knowledge of nutrient-responsive PDH levels and, furthermore, place CobB as a regulator of PDH activity during cell growth in glucose.

Similar experiments were completed to examine KDH activity (Fig. 6B). We demonstrated that cells grown in acetate and glutamine led to higher levels of KDH activity than in those grown in glucose (Fig. 6B). While KDH activity has not been studied in this context before, this finding was in agreement with previous reports (26, 27, 29) and with our observed higher KDH protein levels under similar growth conditions (Fig. 6F). Low KDH activity correlated with low KDH protein levels in glucose (Fig. 6B and F). Additionally, our results demonstrated that CobB deletion leads to increased KDH activity, particularly during growth in glutamine. This increase in activity was not due to increased KDH protein levels (Fig. 6F, compare lanes 5 and 6), highlighting CobB as an important regulator. Therefore, CobB has the most prominent effect on inhibiting enzymatic activity (for either KDH or PDH) when the sole carbon source is upstream of the enzyme studied.

Altogether, these results indicate that CobB has the ability to regulate PDH and KDH activity under different growth conditions, an aspect that has not been previously investigated. PDH and KDH protein levels do not significantly vary between WT and Δ*cobB* deletion strain cells, thus supporting that CobB lipoamidase activity provides a regulatory point at the posttranslational modification level for inhibiting these enzymatic complexes.

## DISCUSSION

Here, we report that sirtuins possess lipoamidase activities in Gram-positive and Gram-negative bacteria. Both CobB and SrtN in *E. coli* and *B. subtilis*, respectively, have been shown to act as lysine deacetylases, and our findings expand their enzymatic functions to include lipoamidase activity. Highlighting the striking similarity of these enzymes between species, SrtN was reported to be able to replace CobB in *Salmonella enterica* and reestablish cell growth (18). Our finding of both conserved lipoamidase activities and substrates significantly expands the knowledge regarding the evolutionary conservation of sirtuin functions.

Evolutionary conservation is a prominent feature of lipoylation. Considering how rare this modification is, it is remarkable that metabolic complexes modified by lipoylation (PDH, KDH, and GCV) are conserved from bacteria to yeast and mammals (2). This led us to propose that lipoylation plays a fundamental role in cellular metabolism and that regulation of lipoylation is crucial for cell survival. In fact, our results suggest that sirtuins can control multiple key regulatory points in central metabolic pathways. This is demonstrated when examining the TCA cycle. Our work has uncovered that CobB and SrtN can inhibit the activities of PDH and KDH. This is relevant when considering that both PDH and KDH regulate carbon entry into the TCA cycle (Fig. 5C). The primary carbon source for the TCA cycle is provided by glucose following glycolysis; carbon enters via the PDH-catalyzed decarboxylation of pyruvate into acetyl-CoA, which is further incorporated as citrate. An alternative carbon source enters the TCA cycle by conversion of glutamine to glutamate, which is incorporated into the TCA cycle as α-ketoglutarate. KDH utilizes α-ketoglutarate in the TCA cycle, converting it to succinyl-CoA. Our study revealed that bacterial sirtuins are positioned at both of these carbon entry points, having the ability to regulate the flow of carbon into this central metabolic process. Our experiments with restricted nutrient availability provided additional support for this. Comparison of cells grown in single-carbon source media, each with distinct entry points into the TCA cycle, established the ability of CobB to differentially respond to nutrient availability. Specifically, CobB inhibits either PDH or KDH when the sole carbon source provided is upstream of that dehydrogenase complex (i.e., glucose for PDH, glutamine for KDH).

In a broader context, our study brings to light an additional enzymatic activity for bacterial sirtuins and, moving forward, it will be interesting to consider the possible interplay between its deacetylase and lipoamidase functions. For example, we observed that CobB deletion had no significant impact on PDH or KDH activities when cells were grown in acetate. It is tempting to speculate that this lack of regulation by CobB is due to its primary role as a deacetylase under this nutrient condition, given that CobB activates acetyl-CoA synthetase via deacetylation, prompting acetyl-CoA synthetase to convert acetate to acetyl-CoA (16, 17). As additional sirtuin activities continue to be discovered, it would be of relevance in future studies to consider how multiple enzymatic activities of one sirtuin are regulated with respect to efficiencies and dynamics. Given the different masses and structures of posttranslational modifications, it is possible that a sirtuin interaction with another protein or factor may assist in achieving an optimal conformation for a certain activity *in vivo*. Additionally, these studies of diverse enzymatic activities highlight the complexity of regulation of cellular metabolic pathways by sirtuins and the importance of understanding their functions in dynamic biological contexts. One such context is pathogenic infection. Given our recent findings that both human and bacterial sirtuins act against viral pathogens (23), it is possible that the identified conserved sirtuin lipoamidase activity and substrates contribute to an ancient mechanism of host defense.

## MATERIALS AND METHODS

### Plasmids and cell culture.

MC4100-wt and MC4100-Δ*cobB E. coli* strains were generated as described previously (23). MC4100-Δ*cobB*-pCobB cells were made by transforming MC4100-Δ*cobB* cells with the His-CobB_6_ Aska(−) plasmid, as described in references 23 and 24. Aska(−) strains were cultured with chloramphenicol (100 µg/ml; Fisher Scientific). pCobB expression was induced by IPTG (Ambion), as described in reference 23. IPTG was added at 0.25 mM when the *E. coli* optical density at 600 nm (OD_600_) was 0.5, followed by 15 min of shaking at 37°C; cells were then cultured in 0.025 mM IPTG at 37°C for 3 h. *B. subtilis* WT and Δs*rtN* strains were a gift from the Dubnau Lab (Rutgers University), and the *srtN* allele was constructed by Jorge Escalante-Semerena (University of Georgia). WT cells were cultured in antibiotic-free media, and the Δs*rtN* deletion strain was grown with spectinomycin (100 µg/ml).

### Measuring lipoylation levels by Western blotting.

Cell density, used to determine protein concentration, was measured based on the OD_600_, and 100 µg of cells was pelleted, lysed in lysis buffer (50 mM Tris [pH 6.8], 2.8% SDS, 10% glycerol, and 0.05% bromophenol blue), and boiled for 10 min. Proteins were resolved by SDS-PAGE and transferred to a nitrocellulose membrane. Antibodies used were anti-lipoic acid (Millipore), anti-maltose binding protein (anti-MBP) and anti-BamA (gifts from the Silhavy Lab), and anti-elongation factor G (anti-EFG; gift from the Dubnau Lab).

### Immunoaffinity purification of Cobb with interacting partners.

IPTG-induced cells were frozen in freezing buffer (20 mM Na-HEPES [pH 7.4], 1/100 bacterial protease inhibitor cocktail) at 100 µl/g of cells, then cryogenically lysed using a Retch MM301 mixer mill (Retch) for 15 cycles of 1.5 min at 30 Hz in a 10-ml jar, as described in references 30 and 31. A small aliquot of ground powder was lysed in 1 ml of cold lysis buffer (20 mM HEPES [pH 7.4], 0.11 M KO-acetate, 2 mM MgCl_2_, 0.1% Tween, 1 µM ZnCl_2_, 1% Triton X-100, 150 mM NaCl, 0.5 mM dithiothreitol, 1:100 bacterial protease inhibitor cocktail [Sigma], 0.5 mM phenylmethylsulfonyl fluoride), and then samples were briefly vortexed and incubated at 4°C with end-over-end rotation for 30 min. Insoluble material was separated from the solubilized fraction by centrifugation at 8,000 × *g* at 4°C for 10 min. For each biological replicate, the soluble fraction was split in half; half was incubated with 10 µg anti-His antibody (Abcam, Inc.), and the other half was incubated with 10 µg of anti-IgG antibody (MP Biomedicals). All samples were incubated for 2 h with rotation at 4°C. Each lysate was then incubated with 0.25 mg of protein A/G magnetic beads (Pierce) for 1 h with rotation at 4°C. Proteins were eluted off the beads by incubation with 100 µl of 1× TEL buffer (247 mM Tris-HCl [pH 8.5], 0.51 mM EDTA, and 2% lithium dodecyl sulfate) at 95°C for 10 min, followed by 10 min of vigorous shaking. Elutions were collected and stored at −20°C until MS analysis was performed, as described in the next section.

### Data-dependent proteomic analysis of Cobb interactions.

The following in-solution digestion proteomic method was based on methods described in references 8 and [Bibr B32][Bibr B33][Bibr B37]), with the modifications specified below. The IP elution fractions (50 µl) were mixed with 400 µl TUD buffer (0.1 M Tris-HCl, 2% sodium deoxycholate, 8 M urea) in Amicon Ultra 0.5 filters (Millipore) and subjected to centrifugation (14,000 × *g*) for 10 min at 20°C. The flowthrough was discarded, and the filter was washed three times with TUD buffer and centrifuged as described above. The filter was then washed with 200 µl of 50 mM ammonium bicarbonate, 2% sodium deoxycholate and centrifuged as above. The filter was transferred to a fresh collection tube, and 100 µl of 50 mM ammonium bicarbonate with 1 µl of MS-grade trypsin (Pierce; 0.5 µg/µl) was added. The filter was shaken for 1 min and then incubated overnight at 37°C. The digested peptides in the eluate were recovered by two washes with 25 µl of water followed by centrifugation, an equal volume of ethyl acetate was added, and the sample was adjusted to 0.5% trifluoroacetic acid (TFA). Following vigorous shaking for 2 min, the sample was centrifuged at 14,000 × *g* for 5 min, and the denser aqueous phase (containing the peptides) was recovered. Peptides were desalted on StageTips using Empore SDB-RPS extraction discs (3M Analytical Biotechnologies). Bound peptides were washed with 50% ethyl acetate–0.5% TFA followed by 0.2% TFA, and then eluted in 50 µl 5% ammonium hydroxide–80% acetonitrile. Samples were concentrated by vacuum centrifugation to near dryness, and then 1% formic acid–4% acetonitrile (ACN) was added to bring the final volume to 9 µl.

Desalted peptides (∼2 µl) were analyzed by nano-liquid chromatography (nLC)-MS/MS using a Dionex Ultimate 3000 nRSLC system coupled directly to a Q Exactive mass spectrometer equipped with an EASY-Spray ion source (Thermo, Fisher Scientific). Peptides were separated by reverse-phase chromatography over a linear 60-min ACN gradient from 4 to 35% mobile phase B at a flow rate of 250 nl/min (mobile phase A was 0.1% formic acid in water, and mobile phase B was 0.1% formic acid in 97% ACN) on an EASY-Spray column (PepMap; 2 µm, 75 µm by 50 cm) with an integrated fused silica emitter. The mass spectrometer was operated in data-dependent acquisition mode with a single acquisition cycle comprising one full-scan mass spectrum (*m/z* 350 to 1,800) in the orbitrap (resolution of 120,000 at *m/z* 200), followed by higher-energy collisional dissociation (HCD) fragmentation of the top 20 most intense precursor ions with dynamic exclusion enabled (exclusion duration, 25 s). MS/MS spectra were extracted and searched using Proteome Discoverer (v.2.1) and SEQUEST (v.1.3) against an *E. coli* protein sequence database (UniProt-Swiss-Prot, 2013_08) appended with common contaminants (4,385 total sequence entries), with the following parameters: full enzyme specificity; 2 missed cleavages; precursor mass tolerance of 10 ppm; fragment mass tolerance of 0.02 Da; static modification of carbamidomethylation of cysteine and dynamic modifications of methionine oxidation and phosphorylation of serine, threonine, and tyrosine residues. For estimation of false discovery rates, the UniProt protein database sequences were reversed and an independent database search was performed. Both forward and reverse SEQUEST peptide spectrum matches were analyzed by Scaffold (v4.4.8; Proteome Software) with a refinement search using the X! Tandem algorithm. Probabilities for peptide spectral matches were calculated using the LFDR algorithm in Scaffold. Protein identifications were filtered in Scaffold at a maximum 1% protein and peptide false discovery rate and a minimum of two unique peptides.

### Quantification of lipoylation by targeted mass spectrometry.

The relative abundance of lipoyl-lysine-containing peptides, previously identified in a data-dependent whole-cell bacterial lysate LC-MS experiment (described above), was measured in whole-cell bacterial lysates using a targeted mass spectrometry approach in an SRM full-scan tandem MS assay. Cells were frozen in freezing buffer (20 mM Na-HEPES [pH 7.4], 1/100 bacterial protease inhibitor cocktail) at 100 µl/g of cells, then cryogenically lysed using a Retch MM301 mixer mill for 10 cycles of 2.75 min at 30 Hz in a 10-ml jar. A small aliquot of ground powder was lysed in ∼50 µl of lysis buffer {50 mM ammonium acetate (pH 6.8), 0.2% RapiGest (Waters Corporation), 10 mM TCEP [tris(2-carboxyethyl) phosphine], 1 mM nicotinamide} in high-performance liquid chromatography (HPLC)-grade water, heated to 70°C. Lysates were heated for 5 min at 70°C and then in two rounds of bath sonication (20 to 30 pulses) and heating at 70°C. The protein concentration was determined by a Bradford assay. Thiols were blocked with *N*-ethylmaleimide (NEM), which was prepared fresh and added at 10 mM, and then the mixture was incubated at 37°C for 1 h. Cysteine was added at 10 mM and the mixture was incubated at 37°C for 15 min to quench excess NEM. Then, 12.5 µl of 0.2 M ammonium bicarbonate (ABC) was added to the reaction mixtures to raise the pH to ∼8.0. Twenty-five micrograms of protein per sample was aliquoted, and mixtures were adjusted to a total volume of 25 µl with 50 mM ABC. GluC enzyme (Thermo, Fisher Scientific) was added in a 1:50 enzyme-to-protein ratio, and the mixture was incubated at 37°C for 3 h, followed by addition of fresh GluC (1:50 ratio) and incubation overnight at 37°C. The samples were acidified to 1% trifluoroacetic acid, incubated at 37°C for 30 min to hydrolyze RapiGest, and then incubated on ice for 30 min. Samples were centrifuged at 4°C at 20,000 × *g* for 10 min. The supernatant was collected and desalted using SDB-RPS StageTips as described in the proteomic analysis section above. Samples were concentrated by vacuum centrifugation, then diluted to ∼1 µg/µl with 1% formic acid–4% ACN. Peptides (∼4 µl) were analyzed using an LC-SRM-MS/MS assay on an LTQ Orbitrap Velos ETD mass spectrometer (Thermo Fisher Scientific), as described in reference 8. Peptides were separated by nLC, as described above, except over a 6-h linear ACN gradient from 4% to 35% B. The mass spectrometer was configured to sequentially isolate targeted precursor ions (precursor window, 2.5 Da) and acquire full-scan MS/MS spectra by collision-induced dissociation (normalized collision energy, 30%) in the ion trap (target value, 10^4^ and 150 ms maximum injection time). Each set of MS/MS acquisitions was followed by a precursor scan in the Orbitrap (resolution, 7,500). Data were imported into Skyline to extract precursor-product ion chromatograms (XICs) and calculate peak areas by using the “targeted” acquisition method and QIT analyzer setting at 0.6-Da resolution. At least four coeluting XICs (dot-product score of 0.95) were used for peak area quantification. Peak picking and integration boundaries were manually verified. Peak areas were summed across XICs, exported to Excel, and normalized across biological replicates (*n* = 3) by using the average chromatographic precursor intensity calculated by RawMeat (Vast Scientific, Inc.). Statistical significance was determined by unpaired, two-tailed *t* tests in Microsoft Excel.

### Purification of His-CobB for *in vitro* assays.

One liter of Aska(−) MC4100 cells was grown to an OD_600_ of 0.5. Next, 100 µM IPTG was added to induce His-CobB expression, followed by 3 h of shaking at 25°C. Cells were washed with phosphate-buffered saline (PBS) and resuspended in 20 ml of lysis buffer (as described above) with DNase and kept on ice for the remainder of the protocol. Cells were vortexed in lysis buffer and then passed through the cell cracker at 12,000 lb/in^2^. Cell debris was pelleted by centrifugation at 8,000 × *g* for 10 min at 4°C. The supernatant was then added to a column containing 2 ml of Ni-NTA–agarose beads (Qiagen) and passed through twice. The column was washed with 10 column volumes of lysis buffer, followed by 10 column volumes of wash buffer (lysis buffer plus 40 mM imidazole). His-CobB was eluted in lysis buffer plus 400 mM imidazole. The elution was dialyzed using a Slide-A-Lyzer dialysis cassette (Thermo Scientific; 10,000 molecular weight cutoff [MWCO]) to remove the low-molecular-weight proteins and buffer exchange for storage buffer (20 mM NaH_2_PO_4_ [pH 7.4], 300 mM NaCl). The manufacturer’s instructions were followed at a temperature of 4°C. Following dialysis, the His-CobB elution fraction was concentrated to ∼400 µl by using a 10,000 MWCO filter (Amicon) and centrifuging for 1 h at 3,000 × *g*. Protein concentration was determined by measuring the *A*_280_, and storage buffer was added to bring the final concentration to 10 mg/ml. Twenty-microliter aliquots were stored at −20°C until ready for use.

### Analysis of CobB activity *in vitro.*

The ability of purified CobB to remove acetyl- and lipoyl-lysine modifications was measured by LC-MS. Synthetic acetyl or lipoyl peptides were generated as described in reference 8 and based on an H3K9 peptide sequence, as this posttranslationally modified peptide sequence had been shown to be readily used in and appropriate for *in vitro* tests of deacylase activities for both mammalian and bacterial sirtuins (8, 10, 38–42). In a 20-µl reaction mixture, 10 µM peptide was incubated with various concentrations of CobB (0.25 to 10 µM), with or without 1 mM NAD^+^ in 50 mM Tris (pH 8.0) for 1 h at 37°C. The reaction was quenched with 25 µl of 2% TFA, and an internal control peptide was added to control for run-to-run variability. Samples were desalted on StageTips by using Empore C_18_ extraction discs (3M Analytical Biotechnologies). Bound peptides were washed once with 0.5% TFA and eluted in 50 µl 0.1% formic acid–80% ACN. Samples were concentrated by vacuum centrifugation to near dryness, then 1% formic acid–4% ACN was added to bring the final volume to 50 µl, and 2 µl of each sample was analyzed by MS as described in reference 8.

### Pyruvate dehydrogenase and *α*-ketoglutarate dehydrogenase activity assays.

PDH and KDH enzymatic activities were measured using Sigma’s pyruvate dehydrogenase (MAK183) and *α*-ketoglutarate dehydrogenase (MAK189) enzymatic activity assays following the manufacturer’s instructions. Cells were grown in LB broth (Miller) before being collected as pellets for analysis. To optimize the PDH assay for *E. coli*, 100-µg cell pellets were lysed in 120 µl lysis buffer for 10 min, and 10 µl of lysate was used per reaction mixture. For *B. subtilis*, reactions were performed as described above, except the mixtures were subjected to bath sonication (5 pulses, repeated 5 times with ice incubation between cycles), followed by an additional 10 min of lysis. For the KDH assay, 1-mg cell pellets were lysed in 300 µl (*E. coli*) or 170 µl (*B. subtilis*) of lysis buffer and bath sonicated as described above. Five microliters (*E. coli*) or 46 µl (*B. subtilis*) of lysate was used per reaction mixture.

### Determining PDH and KDH activities under various nutrient conditions.

MC4100-WT and MC4100-Δ*cobB E. coli* strains were cultured at 37°C with shaking in M63 minimal medium [22 mM KH_2_PO_4_, 51 mM K_2_HPO_4_, 15 mM (NH_4_)_2_SO_4_, and 3 µM FeSO_4_, supplemented with 300 µM thiamine HCl and 1 mM MgSO_4_] with one of the following carbon sources added: 1% glucose (70 mM), 0.4% sodium acetate (60 mM), or 0.4% glutamine (30 mM). Cultures were grown to an OD_600_ of 1, then washed once in PBS, flash-frozen in liquid nitrogen, and stored at −80°C until ready for PDH and KDH activity analysis, which was completed as described above with the following modifications. For the PDH activity, the lysate of the acetate- and glutamine-fed cells was concentrated 4× to detect a signal, and 10 µl of glutamine-fed lysate was used per reaction mixture. All concentration discrepancies were accounted for and normalized during the data analysis.

### Quantifying the impact of nutrient availability on host protein abundances by parallel reaction monitoring-mass spectrometry.

Frozen cell pellets (100 µg) were lysed in 50 µl of 70°C lysis buffer (100 mM Tris-HCl [pH 8.0], 4% SDS, 20 mM TCEP, 20 mM chloroacetamide [CAM], 1 mM EDTA, in HPLC-grade water). Lysates were subjected to three rounds of heating (5 min at 95°C) and bath sonication, and insoluble material was pelleted by spinning at 10,000 × *g* for 5 min. Methanol-chloroform precipitation was performed with the following steps: add 200 µl methanol (LC-MS grade), vortex briefly, add 50 µl chloroform, vortex briefly, add 150 µl HPLC-grade water, vortex briefly, spin for 5 min at 2,000 × *g*, remove the top layer of supernatant, add 150 µl cold methanol, spin for 2 min at 9,000 × *g*, remove supernatant, add 250 µl cold methanol, spin for 2 min at 9,000 × *g*, remove supernatant, briefly dry remaining pellet using vacuum centrifugation (1 to 2 min). The pellet was resuspended in 25 mM of HEPES-KOH, pH 8.2, by bath sonication to ∼0.5 mg/ml. Samples were digested with trypsin at a 1:50 dilution and incubated for 6 h at 37°C. Following digestion, the samples were acidified to 1% TFA, vortexed briefly, incubated at room temperature for 5 min, and spun down at 20,000 × *g* for 10 min. The supernatant was desalted on StageTips using Empore SDB-RPS extraction discs (3M Analytical Biotechnologies). Bound peptides were washed twice with 100 µl of 0.5% TFA and then eluted in 50 µl 5% ammonium hydroxide–80% acetonitrile. Samples were concentrated by vacuum centrifugation to near dryness and then resuspended in 1% formic acid–4% ACN to a final concentration of 1.5 µg/µl.

Peptides (∼2 µl) were analyzed using an LC-parallel reaction monitoring (PRM)-MS/MS assay on a Q Exactive mass spectrometer (Thermofisher Scientific). Peptides were separated by nLC over a 60-min linear ACN gradient from 4% to 35% solvent B, as described above for the proteomic analysis of CobB interactions. The mass spectrometer was configured to perform a scheduled PRM method in which targeted precursor ions (*n* = 49) were sequentially isolated (isolation window, 0.7 Da) and fragmented by HCD fragmentation (resolution, 15,000 at *m*/*z* 200) in a retention time window of 15 min (previously defined from untargeted data-dependent analyses). Each set of full-scan MS/MS acquisitions (loop count, 20) was followed by a precursor ion full scan in the Orbitrap (resolution, 15,000 at *m*/*z* 200). Instrument raw files were imported into Skyline to extract XICs and calculate peak areas by using the Orbitrap “targeted” acquisition setting (15,000 at *m*/*z* 200). At least three coeluting XICs (dot-product score of >0.95) were used for peak area quantification. Peak picking and integration boundaries were manually verified. Peak areas were summed across XICs, exported to Excel, and normalized across biological replicates (*n* = 3) by using the average chromatographic precursor intensity calculated by RawMeat (Vast Scientific, Inc.). Statistical significance was determined with unpaired, two-tailed *t* tests in Microsoft Excel.
