# Supplementary figures and images for: Sirtuin Lipoamidase Activity Is Conserved in Bacteria as a Regulator of Metabolic Enzyme Complexes
Source: mBio. 2017 Sep 12;8(5):e01096-17. doi: 10.1128/mBio.01096-17 (PMC5596343; doi:10.1128/mBio.01096-17)

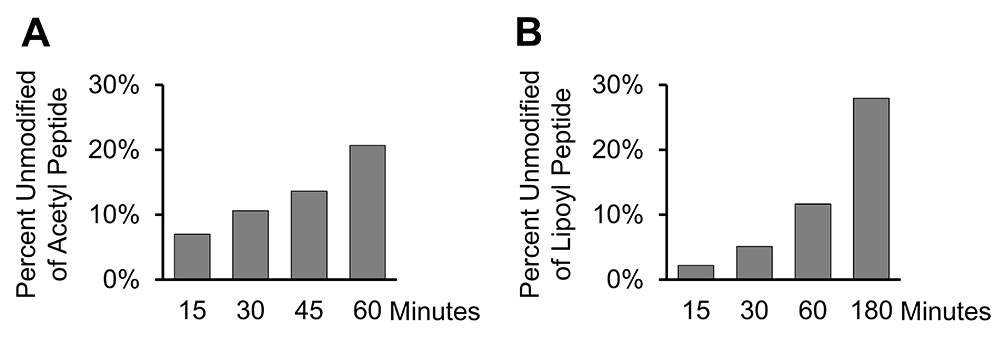

Supplement: FIG S1 [file mbo004173480sf1.tif]
